# The Q system for conditional gene expression is leaky and lacks dynamic range in *C. elegans* neurons

**DOI:** 10.17912/micropub.biology.000573

**Published:** 2022-05-17

**Authors:** Brecht Driesschaert, Liesbet Temmerman

**Affiliations:** 1 Katholieke Universiteit Leuven, Leuven, Belgium

## Abstract

The Q system allows for conditional gene expression in several organisms, including *C. elegans* . We aimed to apply this system in *C. elegans* neurons to obtain temporally-resolved, tissue-specific expression of a fluorescent reporter. We report that, in our hands, there is undesired expression of the reporter in conditions where expression is supposed to be repressed. In addition, in this setup, the signal‑to‑noise ratio of the Q system is unfavorable. We conclude that the Q system is far from optimal in the *C. elegans* nervous system, and advise cautious use.

**Figure 1.**
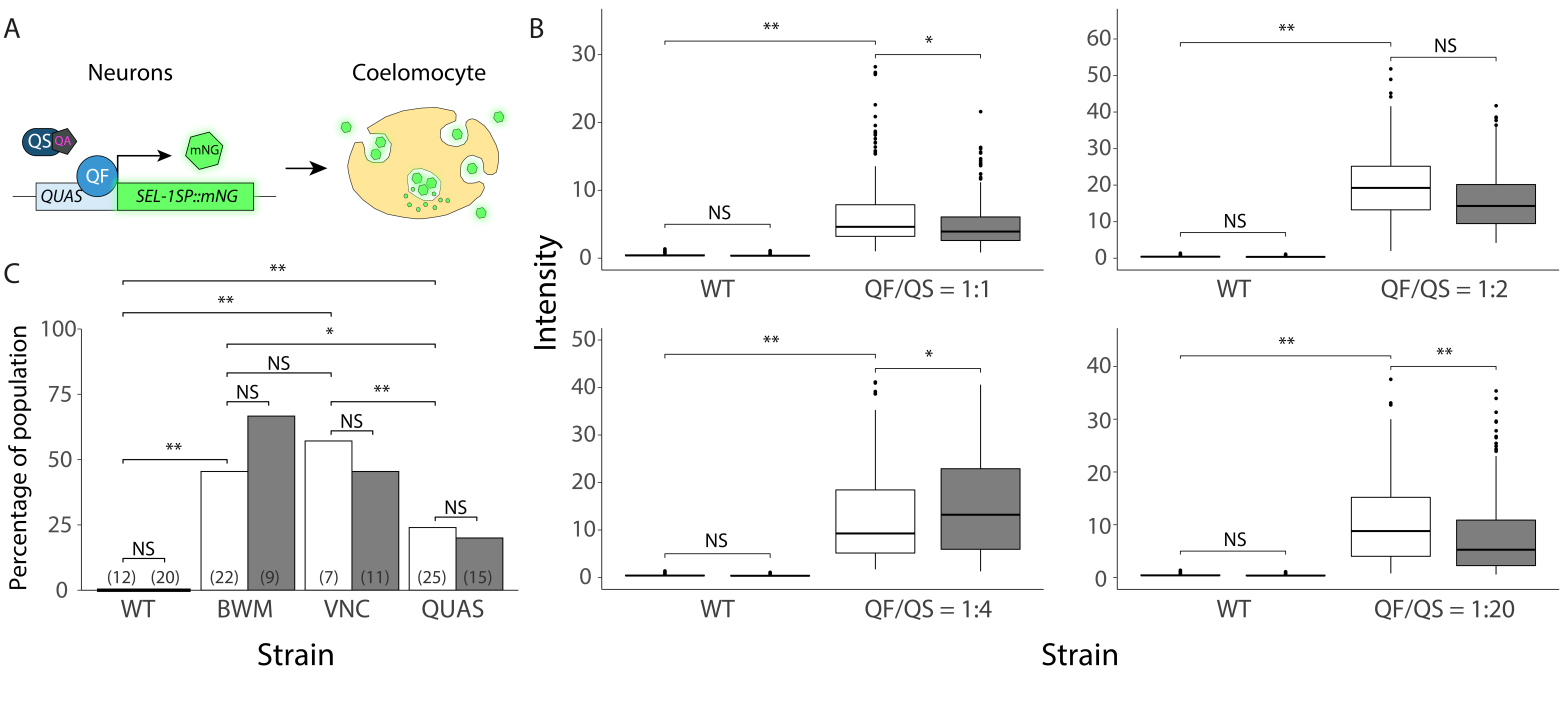
**(A)** Schematic representation of the research objective. The Q system is applied to establish mNeonGreen (mNG) expression and secretion (through the SEL-1 N-terminal signal peptide, SEL-1SP) from *C. elegans* neurons, which will end up being endocytosed and degraded by the coelomocytes. In presence of quinic acid (QA), the transcriptional suppressor (QS) is sequestered, leaving the transcriptional activator (QF) bound to the enhancer sequence ( *QUAS* ), enabling expression of mNeonGreen. Absence of QA permits interaction of QS with QF, blocking transcription (not included in diagram). **(B)** Coelomocyte-specific reporter signal of animals with varying QF/QS ratios, *vs* wild type (WT). The QF/QS ratios indicated on the X‑axis refer to the ratios of the corresponding constructs in the injection mix. Quinic acid absence: white box; presence: grey box. Tukey style box plots: box from Q1 to Q3, line at median value, whisker boundaries at 1.5 interquartile range; dots are individual data points beyond that range. Statistical significance as per type III ANOVA with Bonferroni corrected *p* -value indications ** *p*_adj_ ≤ 0.005, * *p*_adj_ ≤ 0.05 and ‘NS’ *p*_adj_ > 0.05. **(C)** Percentage of worms showing a green fluorescent signal in the target tissue/cells (body wall muscle, ventral nerve cord or coelomocytes) for animals with the Q system expressed in body wall muscle (BWM) or ventral nerve cord (VNC), and a *QUAS* ‑only coelomocyte-targeted reporter strain (QUAS), respectively. For wild‑type animals (WT), all three tissue types were considered. Quinic acid absence: white bar; presence: grey bar. Numbers between parentheses are number of scored worms. Statistical significance as per exact binomial tests with *p* -value indications ** *p*_adj_ ≤ 0.005, * *p*_adj_ ≤ 0.05 and ‘NS’ *p*_adj_ > 0.05.

## Description

The Q system is a genetic tool developed to deliver spatiotemporal control over gene expression (Giles *et al* . 1991; Potter *et al* . 2010; Wei *et al* . 2012). Although it has already been adapted for use in *C. elegans* by Wei *et al* . in 2012, to date, the Q system has not been applied extensively in this nematode. In the relatively few available reports, it is mainly used to constitutively restrict gene expression in a spatial manner ( *e.g.* Schild *et al* . 2014; Schild and Glauser 2015; Jee *et al* . 2016; Tolstenkov *et al* . 2018; Chiyoda *et al* . 2021), while but a handful of studies also explore the temporal aspect of the system (Matus *et al* . 2015; Yuan *et al* . 2016; Cottee *et al* . 2017; Hoang and Miller 2017). We aimed to apply this tool in the *C. elegans* nervous system to gain both spatial and temporal control over expression of a gene encoding a reporter protein that is targeted to the secretory pathway. Despite our efforts, we here report that in our hands, the Q system is not suitable for application in the neurons due to a lack of dynamic range.

We initially turned to the Q system based on an interest in obtaining temporally-resolved, neuron-specific secretion of a reporter into the pseudocoel, from where it can be taken up and degraded by the coelomocytes (Altun and Hall 2009) (Figure 1A). We relied on the pan-neuronal *rab* - *3* promoter (Stefanakis *et al* . 2015) to express a fusion protein consisting of mNeonGreen (Hostettler *et al* . 2017) and the N-terminal signal peptide of SEL-1, which targets the reporter protein via the secretory pathway into the pseudocoel (Grant and Greenwald 1997). In these reporter animals one would expect to observe fluorescent signal in source and sink cells: neurons and coelomocytes, the latter accumulating the signal through endocytosis of pseudocoelomic content (Altun and Hall 2009).

In this setup, we aimed to use the Q system to gain temporal control over reporter production. This binary conditional expression system consists of a transcriptional activator QF, a transcriptional suppressor QS and an enhancer sequence *QUAS* , to be placed upstream of the sequence of interest, here encoding to‑be‑secreted mNeonGreen (Figure 1A). Both the transcriptional activator QF and suppressor QS are expressed tissue-specifically, in this case using the pan‑neuronal *rab* - *3* promoter. In the absence of quinic acid, the activator QF binds the enhancer sequence *QUAS* , but is unable to drive expression of the gene of interest because of the presence of the suppressor QS. Upon addition of quinic acid, the suppressor QS is (reversibly) sequestered, which allows for the expression of the gene of interest, driven by the activator QF bound to the enhancer sequence *QUAS* (Potter *et al* . 2010; Wei *et al* . 2012). This means that in our case, a green fluorescent signal should be observed in neurons and coelomocytes under positive control conditions (24h quinic acid treatment), but not in negative control conditions (permanent absence of it).

To examine this, synchronized populations of wild‑type (N2) or reporter larvae (L1, 24h after the egg was laid) were distributed over *E. coli* ‑seeded Nematode Growth Medium plates containing quinic acid, *versus* negative control plates to which no small molecule was added. After 24h, the L4‑stage animals were imaged, and the pixel intensity of the green fluorescent signal in the coelomocytes was quantified using ImageJ. This revealed that there is considerable leaky expression when employing the Q system in the neurons using *rab* - *3p* , *i.e.* the green fluorescent signal could already be detected in the coelomocytes of reporter animals at high intensities in the absence of quinic acid (Figure 1B). We attempted to overcome this issue by increasing the amount of suppressor QS *versus* activator QF, analogous to Potter *et al* . 2010. Unfortunately, this did not affect the outcome: for each QF/QS ratio tested (1:1, 1:2, 1:4 and 1:20; Figure 1B and Table 1, Exp. #1‑3), the reporter negative control condition (no quinic acid) was significantly different from the wild-type N2 condition (no Q system), indicating the undesired production of mNeonGreen in the absence of quinic acid, or the system’s leakiness. In addition, the signal‑to‑noise ratio of the Q system is unfavorable when applied in neurons, *i.e.* for reporter animals, none of the negative control *vs* quinic acid treatment conditions differ substantially (Figure 1B), making the Q system inapplicable when driven from the pan-neuronal *rab* - *3p* .

Despite some leakiness, others have been able to work with the Q system in the nervous system of *Drosophila melanogaster* (Potter *et al* . 2010) and *C. elegans* (Wei *et al* . 2012). Therefore, we performed a follow‑up experiment including published reagents of the latter report (kindly provided by the Shen lab, Stanford University, Stanford, California, USA; Table 1, Exp. #4). Here, we counted the number of animals containing any reporter signal in the target tissue, irrespective of intensity. For both strains and irrespective of quinic acid treatment, a similar number of animals contained reporter signal (Figure 1C). Together, these findings could point towards an unknown factor interfering with the susceptibility of the neurons (or other tissues, albeit possibly less pronounced) to the Q system in our hands. Alternatively, the lack of dynamic range might originate from an actual incompatibility between the Q system and the *C. elegans* nervous system. If so, this could be explained from two perspectives: *(1)* the Q system might be inherently leaky to some extent, but it may be harder to activate in *C. elegans* neurons compared to other cell types, or *(2)* the Q system may be excessively leaky in neurons.

Our data seem to support the first option, since both published reagents (Figure 1C) display a similar proportion of worms with green fluorescence in the target tissue in the negative control condition (no quinic acid), which is higher than for the wild-type N2 condition (no Q system, no GFP observed; Figure 1C). So the Q system indeed appears to be inherently leaky, but not excessively in neurons. However, the second option cannot be entirely ruled out, since our data do not provide information on the actual signal intensities of the target tissues. While we observe similar proportions of worms with leaky signal, there may yet be strain-related differences in signal intensities in these animals, which would unveil possible tissue-related differences in Q system leakiness. In summary, both options can be supported by our data, and further research would be required if one wanted to explore this in more detail.

The first option could result from an inability of quinic acid to reach the neuronally expressed suppressor QS. It is certainly not the case that *C. elegans* neurons are generally inaccessible to small molecules provided in the medium, since auxin (indole‑3‑acetic acid) for example has been successfully used to control protein degradation in these cells *in vivo* ( *e.g.* McDiarmid *et al* . 2020; Ashley *et al* . 2021; Zhang *et al* . 2021). Compared to auxin, however, quinic acid is more hydrophilic, which may impede passive diffusion into (certain) cells. Supporting this notion, diffusion of quinic acid into central brain neurons is hampered by the blood‑brain barrier of *D. melanogaster* (Riabinina *et al* . 2015; Riabinina and Potter 2016). While *C. elegans* does not have a blood‑brain barrier, it may be possible that its neurons are less permeable to passive diffusion of small molecules than cells of other tissues. Alternatively, there may be differences in active uptake of small molecules depending on the molecule and cell identity, mechanisms of which are unknown – at least for quinic acid (and auxin) – in *C. elegans* today.

In the context of the alternative perspective of excessive leakiness, we evaluated reporter signal of a strain that lacks both the activator QF and the suppressor QS, containing only the to‑be‑secreted fluorescent mNeonGreen reporter DNA code under control of the enhancer sequence *QUAS* (Table 1, Exp. #5). Green fluorescence can be observed in coelomocytes in a significantly higher proportion of worms in the negative control condition (no quinic acid) of this *QUAS* ‑only strain compared to the wild-type N2 condition (no Q system, no mNeonGreen observed; Figure 1C), indicating the possible presence of endogenous transcription factors that are able to bind the enhancer sequence *QUAS* and drive expression in the absence of quinic acid. Excessive leakiness of the Q system in the nervous system would imply that the nuclear environment of neural cells differs from other cells in such a way that it enables expression driven by the enhancer sequence *QUAS* in the absence of quinic acid to a greater extent compared to other tissues. Still, since the negative control conditions (no quinic acid) of the published reagents contain a significantly higher proportion of worms showing green fluorescence in the target tissue compared to the negative control condition (no quinic acid) of the *QUAS* ‑only strain, possible endogenous transcription factor activities can only partly explain the leakiness of the Q system. More research would be required to explore and identify other possible (tissue-specific) factors that might be involved, for example at the level of the activator-suppressor interaction, rather than at the level of the enhancer sequence *QUAS* .

At the moment, it is unclear whether our observations are due to experimental conditions, or due to characteristics of the worm’s nervous system. In any case, it seems that yet-unknown particularities of the *C. elegans* nervous system (possibly in interaction with experimental setup) are to be taken into account in genetic tool development. Overall, we advise caution when applying the Q system in this tissue, and suggest turning to other conditional expression systems for use in the *C. elegans* nervous system.

## Methods

All strains (Table 2) were maintained at 20°C on *E. coli* OP50‑seeded Nematode Growth Medium (NGM) according to standard procedures (Stiernagle 2006).

Animals of the in-house generated *QUAS* ‑only coelomocyte-targeted reporter strain with QF/QS = 0:0 were injected with a single DNA construct: *(1)* *QUAS::SEL* - *1SP::mNeonGreen* . Animals of in-house generated strains with QF/QS = 1:1 were injected with a mix of two DNA constructs: *(1)* *rab* - *3p::QS::SL2::QF* and *(2)* *QUAS::SEL* - *1SP::mNeonGreen* . The other in-house generated reporter animals (QF/QS = 1:2, 1:4 and 1:20) were injected with mixes of three constructs: *(1)* *rab* - *3p::QS, (2) rab* - *3p::QF* and *(3)* *QUAS::SEL* - *1SP::mNeonGreen* . QF/QS ratios refer to the ratios of the corresponding constructs in the injection mix. The sequences of the enhancer *QUAS* , the transcriptional activator QF and the transcriptional repressor QS originate from the plasmids XW12, XW08 and XW09, respectively (obtained from AddGene).

We tested the Q system according to Wei *et al* . 2012, with some adjustments (in consultation with Wei *et al.* , personal communication) to optimize the assay.

*Quinic acid plates* – A quinic acid working solution was prepared by adding 720 µL M9 buffer and 1440 µL NaOH (5 M, Sigma‑Aldrich, Catalog # S5881) to 5400 µL of a fresh quinic acid stock solution (300 mg/mL, D‑(‑)‑quinic acid [Sigma‑Aldrich, Catalog # 138622] in Milli‑Q water, stored at 4 °C in the dark). The pH of the working solution was 6.0 ‑ 7.5 as verified using pH‑indicator strips (Dosatest®, pH 1.0 – 12.0 [VWR Chemicals, Art. Code 35300.606]) and if required, the solution was brought in this pH range by topping up with NaOH (5 M, Sigma‑Aldrich, Catalog # S5881). The quinic acid working solution was also kept in the dark at 4 °C. To prepare quinic acid plates, 353 µL working solution were pipetted dropwise onto each *E. coli* ‑seeded NGM plate (55 mm diameter), in a sterile way. The plates were then shielded from light, and left to dry in a sterile environment.

*Quinic acid assay* – Prior to the assay, for each strain a synchronized population of worms was obtained by allowing 20 day‑2 adults (pre‑synchronized by picking L4‑stage worms in advance, no bleaching) to lay eggs on an *E. coli* ‑seeded NGM plate (55 mm diameter) for approximately 2‑3 hours. The adult worms were removed from the plate and the eggs were allowed to hatch over a period of 24 hours. About a half of the larvae were then transferred to two quinic acid plates by chunking two quarters of the original plate. The remainder of the population was kept as the negative control plate. Quinic acid plates were stored in a separate box wrapped in tinfoil to shield them from light. After approximately 24 hours, L4‑stage worms were imaged.

*Image acquisition* – About 25 worms per condition (Table 1) were mounted onto 2% agarose (Sigma‑Aldrich, Catalog # A9539) pads containing 10 µL M9 buffer and 10 µL tetramisole solution (10 mM, (-)‑tetramisole hydrochloride [Sigma‑Aldrich, Catalog # L9756]), after which a cover glass was placed on top. Images were made using a Leica DM6 B microscope with a 10X objective and GFP filter, using its LAS X software.

*Quantification and data analysis* – Images (16 bit) of reporter animals (QF/QS ratios 1:1, 1:2, 1:4 and 1:20) and wild types (Table 1, Exp. #1‑3) were processed using ImageJ (NIH) by delineating the coelomocytes and measuring the mean pixel intensity in this region of interest. Statistical analysis was performed on pooled data from multiple replicates of the same QF/QS ratios (multiple strains per ratio and/or multiple independent repeats per strain, see Table 1 for details) to compare negative control with quinic acid treatment conditions as well as wild types with reporter animals using a type III ANOVA with Bonferroni correction. Images of the published reagents (body wall muscle and ventral nerve cord) and *QUAS* ‑only coelomocyte-targeted reporter strain (Table 1, Exp. #4‑5) were scored in a binary manner based on observation of a green fluorescent signal in the target tissue/cells (body wall muscle, ventral nerve cord or coelomocytes), respectively, irrespective of the intensity (YES: green fluorescent signal observed in the target tissue; NO: the target tissue shows no green fluorescence at all). For wild‑type animals, all three tissue types were considered. Statistical analysis was performed to compare negative control with quinic acid treatment conditions as well as to reciprocally compare all negative control conditions using exact binomial tests. R software (version 3.6.3) and Adobe Illustrator were used to make Figure 1.

**Table 1. Number of animals used in individual experiments.** Noted as: x ; y, with x = number of animals in negative control condition, and y = number of animals in quinic acid treatment condition; ‘-’ = not included in experiment.

**Table:** 

**Wild-type strain**						
**QF/QS ratio**	**Strain name**	**Exp. #1**	**Exp. #2**	**Exp. #3**	**Exp. #4**	**Exp. #5**
0:0	N2	14 ; 20	25 ; 11	20 ; 18	12 ; 20	22 ; 20
**In-house generated strains**						
**QF/QS ratio**	**Strain name**	**Exp. #1**	**Exp. #2**	**Exp. #3**	**Exp. #4**	**Exp. #5**
0:0	LSC1879	-	-	-	-	25 ; 15
1:1	LSC1953	23 ; 18	32 ; 20	0 ; 25	-	-
	LSC1954	22 ; 17	-	-	-	-
1:2	LSC1965	30 ; 23	18 ; 27	27 ; 26	-	-
1:4	LSC1966	29 ; 24	-	22 ; 16	-	-
	LSC1967	22 ; 20	-	-	-	-
	LSC1968	27 ; 27	-	-	-	-
1:20	LSC1978	-	8 ; 9	33 ; 27	-	-
	LSC1979	-	25 ; 23	23 ; 10	-	-
	LSC1980	-	25 ; 20	-	-	-
	LSC1981	-	18 ; 19	-	-	-
** Published reagents (Wei *et al* . 2012; received from Shen lab, Stanford University, Stanford, California, USA) **						
**QF/QS ratio**	**Strain name**	**Exp. #1**	**Exp. #2**	**Exp. #3**	**Exp. #4**	**Exp. #5**
1:1	TV10187	-	-	-	7 ; 11	-
1:1	TV11503	-	-	-	22 ; 9	-

## Reagents

**Table 2. Strains used in this report.** Details include strain name and genotype. N2 wild types are available via the CGC (Minnesota, USA).

**Table:** 

**Wild-type strain**	
**Strain name**	**Genotype**
N2	Bristol wild‑type strain
**In-house generated strains**	
**Strain name**	**Genotype**
LSC1879	*lstEx1041[QUAS::Δpes* - *10::sel* - *1SP::mNeonGreen 5 ng/µL;* *myo* - *2p::mCherry::unc* - *54 3’ UTR 1 ng/µL]*
LSC1953	*lstEx1063[rab* - *3p::QS::SL2::QF::unc* - *54 3’ UTR 5 ng/µL;* *QUAS::Δpes* - *10::sel* - *1SP::mNeonGreen 5 ng/µL;* *myo* - *2p::mCherry::unc* - *54 3’ UTR 1 ng/µL]*
LSC1954	*lstEx1063[rab* - *3p::QS::SL2::QF::unc* - *54 3’ UTR 5 ng/µL;* *QUAS::Δpes* - *10::sel* - *1SP::mNeonGreen 5 ng/µL;* *myo* - *2p::mCherry::unc* - *54 3’ UTR 1 ng/µL]*
LSC1965	*lstEx1067[rab* - *3p::QF::rab* - *3 3’ UTR 7.63 ng/µL;* *rab* - *3p::QS::rab* - *3 3’ UTR 14.57 ng/µL;* *QUAS::Δpes* - *10::sel* - *1SP::mNeonGreen 5.24 ng/µL;* *myo* - *2p::mCherry::unc* - *54 3’ UTR 1 ng/µL]*
LSC1966	*lstEx1068[rab* - *3p::QF::rab* - *3 3’ UTR 7.63 ng/µL;* *rab* - *3p::QS::rab* - *3 3’ UTR 29.13 ng/µL;* *QUAS::Δpes* - *10::sel* - *1SP::mNeonGreen 5.24 ng/µL;* *myo* - *2p::mCherry::unc* - *54 3’ UTR 1 ng/µL]*
LSC1967	*lstEx1068[rab* - *3p::QF::rab* - *3 3’ UTR 7.63 ng/µL;* *rab* - *3p::QS::rab* - *3 3’ UTR 29.13 ng/µL;* *QUAS::Δpes* - *10::sel* - *1SP::mNeonGreen 5.24 ng/µL;* *myo* - *2p::mCherry::unc* - *54 3’ UTR 1 ng/µL]*
LSC1968	*lstEx1068[rab* - *3p::QF::rab* - *3 3’ UTR 7.63 ng/µL;* *rab* - *3p::QS::rab* - *3 3’ UTR 29.13 ng/µL;* *QUAS::Δpes* - *10::sel* - *1SP::mNeonGreen 5.24 ng/µL;* *myo* - *2p::mCherry::unc* - *54 3’ UTR 1 ng/µL]*
LSC1978	*lstEx1074[rab* - *3p::QF::rab* - *3 3’ UTR 5 ng/µL;* *rab* - *3p::QS::rab* - *3 3’ UTR 100 ng/µL;* *QUAS::Δpes* - *10::sel* - *1SP::mNeonGreen 5 ng/µL;* *myo* - *2p::mCherry::unc* - *54 3’ UTR 1 ng/µL]*
LSC1979	*lstEx1074[rab* - *3p::QF::rab* - *3 3’ UTR 5 ng/µL;* *rab* - *3p::QS::rab* - *3 3’ UTR 100 ng/µL;* *QUAS::Δpes* - *10::sel* - *1SP::mNeonGreen 5 ng/µL;* *myo* - *2p::mCherry::unc* - *54 3’ UTR 1 ng/µL]*
LSC1980	*lstEx1074[rab* - *3p::QF::rab* - *3 3’ UTR 5 ng/µL;* *rab* - *3p::QS::rab* - *3 3’ UTR 100 ng/µL;* *QUAS::Δpes* - *10::sel* - *1SP::mNeonGreen 5 ng/µL;* *myo* - *2p::mCherry::unc* - *54 3’ UTR 1 ng/µL]*
LSC1981	*lstEx1074[rab* - *3p::QF::rab* - *3 3’ UTR 5 ng/µL;* *rab* - *3p::QS::rab* - *3 3’ UTR 100 ng/µL;* *QUAS::Δpes* - *10::sel* - *1SP::mNeonGreen 5 ng/µL;* *myo* - *2p::mCherry::unc* - *54 3’ UTR 1 ng/µL]*
** Published reagents (Wei *et al* . 2012; received from Shen lab, Stanford University, Stanford, California, USA) **	
**Strain name**	**Genotype**
TV10187	*wyEx4048[unc* - *4p::QF::SL2::mCherry(XW08) 5 ng/µL;* *unc* - *4p::QS::SL2::mCherry(XW25) 5 ng/µL;* *QUAS::Δpes* - *10::GFP(65C)(XW12) 5 ng/µL;* *odr* - *1p::RFP 60 ng/µL]*
TV11503	*wyEx4697[myo* - *3p::QF 10 ng/µL;* *myo* - *3p::QS::SL2::mCherry 10 ng/µL;* *QUAS::Δpes* - *10::GFP 10 ng/µL;* *odr* - *1p::dsRed 60 ng/µL;* *pBluescript 50 ng/µL]*
